# Evaluation of Carbon Dioxide-Based Urethane Acrylate Composites for Sealers of Root Canal Obturation

**DOI:** 10.3390/polym12020482

**Published:** 2020-02-21

**Authors:** Hao-Hueng Chang, Yi-Ting Tseng, Sheng-Wun Huang, Yi-Fang Kuo, Chun-Liang Yeh, Chien-Hsin Wu, Ying-Chi Huang, Ru-Jong Jeng, Jiang-Jen Lin, Chun-Pin Lin

**Affiliations:** 1 Graduate Institute of Clinical Dentistry, School of Dentistry, National Taiwan University, Taipei 100, Taiwan; changhh@ntu.edu.tw (H.-H.C.); yelsaint@gmail.com (S.-W.H.); franca_kay@hotmail.com (Y.-F.K.); staryeh0524@gmail.com (C.-L.Y.); 2 Department of Dentistry, National Taiwan University Hospital, Taipei 100, Taiwan; 3 Institute of Polymer Science and Engineering and Advanced Research Center for Green Materials Science and Technology, National Taiwan University, Taipei 100, Taiwan; kop11239@gmail.com (Y.-T.T.); chwuoliver@gmail.com (C.-H.W.); inzmehuang@gmail.com (Y.-C.H.); 4 Department of Materials Science and Engineering, National Chung Hsing University, Taichung 400, Taiwan

**Keywords:** root canal obturation, composites, urethane acrylates, nanoscale silicate platelets, carbon dioxide-based resins

## Abstract

A new root canal sealer was developed based on urethane acrylates using polycarbonate polyol (PCPO), a macrodiol prepared in the consumption of carbon dioxide as feedstock. The superior mechanical properties and biostability nature of PCPO-based urethane acrylates were then co-crosslinked with a difunctional monomer of tripropylene glycol diarylate (TPGDA) as sealers for resin matrix. Moreover, nanoscale silicate platelets (NSPs) immobilized with silver nanoparticles (AgNPs) and/or zinc oxide nanoparticles (ZnONPs) were introduced to enhance the antibacterial effect for the sealers. The biocompatibility and the antibacterial effect were investigated by Alamar blue assay and LDH assay. In addition, the antibacterial efficiency was performed by using *Enterococcus faecalis* (*E. faecalis*) as microbial response evaluation. These results demonstrate that the PCPO-based urethane acrylates with 50 ppm of both AgNP and ZnONP immobilized on silicate platelets, i.e., Ag/ZnO@NSP, exhibited great potential as an antibacterial composite for the sealer of root canal obturation.

## 1. Introduction

**Figure 1:**
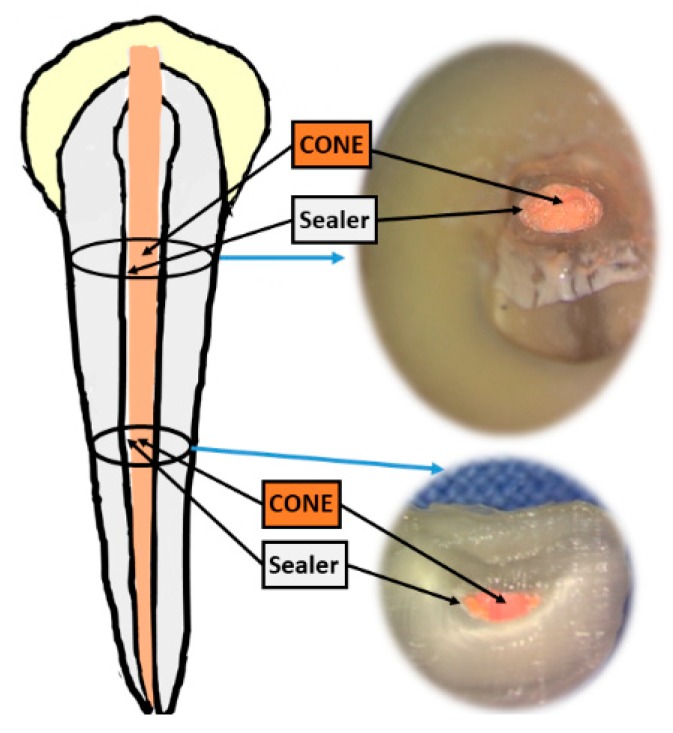
The illustration of filling materials for root canal therapy.

A tooth with damaged or injured dental pulp requires root canal therapy to cure or prevent apical periodontitis. However, the root canal system has complex internal anatomy with high prevalence [1]. The successful root canal therapy includes correct diagnosis, adequate debridement, and dense filling of three-dimensional space. The root canal obturation is to fill up the root canal and to prevent microorganisms from re-entering into the canal system [2]. Obturation is the method used to fill and seal a cleaned and shaped root canal using core filling materials and root canal sealer. The root canal filling materials are usually composed of cone and sealer (Figure 1). Gutta-percha (GP) cone is the most commonly used material for the obturation of the root canal therapy. Due to the insufficient dentinal adhesion to GP cone, the uses of endodontic sealers are required to provide cohesive strength between core material interface and root canal dentin wall to hold the obturation material together [3]. The root canal sealers are the filling materials that cover all areas of the canal, while acting as lubricants to reduce the friction resistance between the cone material and the canal wall [4,5]. As a result, the key factor to the success of root canal therapy relies on the choice of the appropriate filling material that can completely cover the root canal to prevent the infection.

Typical root canal sealers are composed of zinc-oxide eugenol, calcium hydroxide, or glass ionomer. Many studies have suggested that the conventional root canal sealers are easily dissolved [6] and have no dentinal adhesion [7]. Recently, bioceramics such as tricalcium silicate-, dicalcium silicate-, calcium phosphates-, colloidal silica-, or calcium hydroxide-based root canal sealers were developed [8,9]. The moisture within the dentinal tubules was utilized to solidify the calcium silicate hydrate phase [10]. Subsequently, hydroxyapatite was precipitated within the hydrate phase to produce a composite-like structure with reinforcing effects and good sealing ability. Moreover, the pH of the bioceramic sealer during the setting process was usually higher than 12, which increased its bactericidal properties under such circumstances. As compared to the typical root canal sealers, the presence of water is usually a requirement in the canal space for the hydration reaction in order to enable the materials’ solidification in the bioceramic sealers such as calcium silicate-based endodontic sealers [11]. However, the difficulty on controlling the precise water content in the bioceramic sealers would result in uncertain setting times along with microhardness. Furthermore, Pawar et al. [12] also suggested that the use of the new bioceramic sealer usually exhibits a lower bond strength than the commonly used root filling materials such as AH-Plus^®^ during the push-out bond strength test. The development of composites for dental-clinical performance still a challenging topic nowadays.

Apart from that, a series of novel endodontic sealers has been derived from synthetic polymers, such as epoxy-, methacrylate-, and silicon-based resins. [13,14,15,16] Moreover, urethane-dimethacrylate-based resins were also developed in order to achieve adhesion with dentin and resin properties [13,14]. As one of the key components in the success of advanced technologies, the urethane-based resins can be easily obtained from a rapid formation between the isocyanate chemistry and raw materials with active hydrogens. A large variety of properties can be tailored in the biomedical applications such as wound dressings, artificial organs, and tissue scaffolds [17]. Although the improved bonding strength between the sealers and dentin has been realized, the adhesive bonding with cone materials is still a challenging issue. The gaps along with the sealer-dentin interfaces would attribute to the polymerization shrinkage of the sealer, leading to the re-infection result after the root canal therapy.

The incorporation of nanoscale antibacterial materials is one of the solutions to produce an antibacterial root canal sealer material. The nanoscale silicate platelets (NSP) previously developed by our research group were used as supports for nanoparticle dispersion, such as silver nanoparticles (AgNPs) [18,19]. The nanometer-thin silicate platelets were intended for surface interactions in order to achieve NSPs immobilized with AgNPs (Ag@NSP) [20]. In fact, Ag@NSP demonstrated excellent antimicrobial activities against common bacteria. Their antibacterial activities depends heavily on the particle size of AgNPs along with the weight ratios of the mobilized AgNPs on NSPs [21]. Moreover, Ag@NSP with low cytotoxicity would further promote wound healing [22]. Apart from that, ZnONPs are employed extensively in a variety of areas such as health products, cosmetics, and catalyst industry owing to their optoelectronic properties, high catalytic efficiency, and antibacterial activities [23,24]. A considerable enhancement on the antibacterial bioactivity toward microorganisms along with biocompatibility to human cells can be achieved by ZnONPs [25]. Therefore, ZnONPs were also immobilized on the nanometer-thin silicate platelets for further enhancing antibacterial properties. Based on the above, nanohybrids such as Ag@NSP, ZnO@NSP or Ag/ZnO@NSP are have great potential as antibacterial agents for the root canal applications.

The purpose of this study was to develop a novel root canal sealer, using a biocompatible resin matrix with NSPs as antibacterial components. The urethane resins were first prepared from the aliphatic polycarbonate polyol (PCPO), which is a macrodiol prepared in the consumption of carbon dioxide [26,27]. The urethane acrylates prepared from PCPO exhibited not only excellent resistance to hydrolysis and good mechanical properties but significant improvement in biostability and nontoxicity in many cases [28,29]. It was also reported that the NSPs immobilized with nanoparticles such as AgNPs could effectively prevent the nanoparticles from agglomeration into Ag clusters, while exhibiting antimicrobial activity through dramatically decreasing glucose uptake and hindering adenosine triphosphate (ATP) synthesis for microbial growth [21]. As a result, the root canal sealers were then prepared by urethane acrylate (UA) composites comprising the immobilized nanoparticles on nanoscale platelets for the root canal obturation evaluations.

## 2. Materials and Methods

### 2.1. Synthesis of Urethane-acrylate (UA)

**Figure 2:**
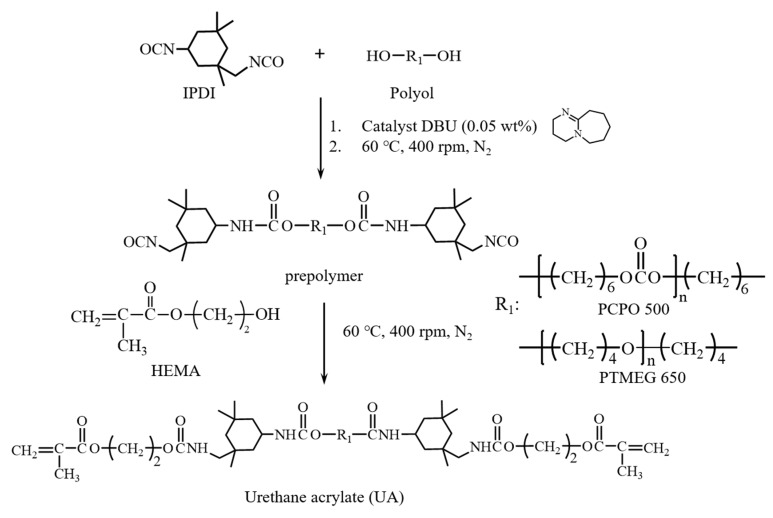
The preparation of urethane acrylate (UA).

As shown in Figure 2, UAs were prepared from the reaction between the isocyanate groups of isophorone diisocyanate (IPDI) in the presence of 1,8-diazabicyclo[5.4.0]undec-7-ene (DBU) as catalyst, and hydroxyl groups of polyols to achieve urethane prepolymer end-capped with isocyanates, followed by the addition of hydroxyethylmethacrylate (HEMA). In this study, UA prepared from polyols by using polytetramethylene ether glycol with a Mw of 650 (PTMEG 650) is denoted as UAT65, while UA prepared from polycarbonate polyol with a Mw of 500 (PCPO 500) is denoted as UAC50. The introduction of acrylate group was prepared from using HEMA as reagent in the molar ratio (2/1/1) of IPDI/polyol/HEMA. The synthesis of UAs was monitored by using IR spectrum (Jasco 4600 FT-IR Spectrophotometer with a Jasco ATR Pro 450-S accessory, Jasco, Tokyo, Japan). The molecular weights were measured by using gel permeation chromatography (GPC) with samples dissolved in 1 wt % THF. (Waters chromatography system, two Waters Styragel linear columns, and polystyrene as the standard with Mw 104, 2560, 7600, 18,000, and 37,900 by using THF as eluent at the flow rate of 1 mL/min.)

### 2.2. Preparation of Antibacterial Sealer Based on UA

The UA samples (UAT65 and UAC50) were dissolved in acetone at 80 °C and mixed with nanoscale silicate materials (Ag@NSP, ZnO@NSP, or Ag/ZnO@NSP) to prepare UA-based composites. The preparation and characterization of Ag@NSP, ZnO@NSP, and Ag/ZnO@NSP were performed according to the literature reported by our groups [21]. The sealers were then prepared by the introduction of a diluent, tripropylene glycol diacrylate (TPGDA), and an initiator containing camphorquinone (CQ) as a photosensitizer with ethyl-4-dimethylaminobenzoate (EDMA) for binary initiation processes with a ratio of CQ/EDMAB = 1/2 (*w/w*), together with 0.5 wt % azobisisobutyronitrile (AIBN) for ternary initiation processes. The characterizations of composites were carried out by using the thermogravimetric analysis (TGA; TGA Q50 TA instrument, TA Instrument, New Castle, DE, USA) to investigate the inorganic contents. For the curing process, the sealer samples were first cast into Teflon molds in the dimension of 20-mm long, 2-mm wide, and subsequently cured with a light emitting diode curing unit at an intensity of 800 mW/cm^2^ for 40 s from the coronal aspect (SmartLite, Dentsply, PA). The degrees of curing conversions were investigated by using FT-IR to calculate the peak area transition at 1638 cm^−1^ of aliphatic C=C double bond of acrylate group according to the following equation (1) [30]:

(1) $$\mathrm{Degree}\;\mathrm{of}\;\mathrm{conversion}\;\%\;(\mathrm{DC}\%)=\left[1-\frac{{\left[A_{1638}\right]}_{\mathrm{after}\;\mathrm{curing}}}{{\left[A_{1638}\right]}_{\mathrm{before}\;\mathrm{curing}}}\right]\times 100\%$$

### 2.3. Curing Condition and Depth Test

**Figure 3:**
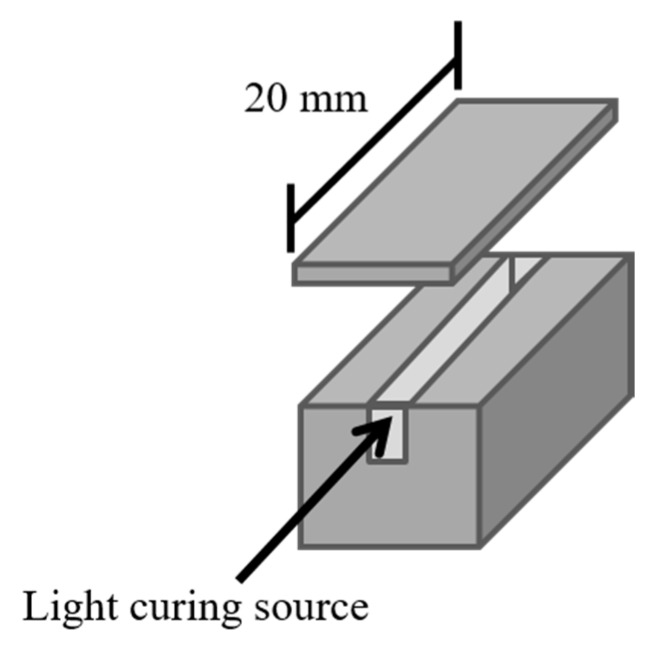
The test method for curing depths according to ISO 4049:2009 [33].

To evaluate the feasibility of the sealers for root canal, the flow and viscosity tests were carried out for UA resins according to ISO 6876:2001. Based on the specifications, the mixture (0.05 mL) of UA resin and TPGDA (UAT65/TPGDA or UAC50/TPGDA) was placed on the center of glass plate using a graduated syringe. After the initial mixing for 180 s (±5 s), another glass plate was placed on top of the sealer, following a weight providing a total mass of 120 g (±2 g). After 10 min, the weight was removed in order to measure the maximum and minimum diameter of the compressed disks of the sealers [31]. Apart from that, the viscosities of the sealers were measured by using a syringe-based viscometer based on Instron 3360 series universal testing system [32]. In Figure 3, the measurement of maximal curing increment thickness for resin composites was conducted by using a ISO 4049:2009 method [33]. The curing process was performed with a 2 × 2 × 20 mm re-usable stainless-steel mold with a top cover to prevent materials’ leakage. After curing process under a light source of halogen lamp for 40 s, all samples were kept in saturated humidity at 37 °C for 24 h to measure the curing depths. The mechanical properties were also measured by using the Universal Testing Instrumentals according to ASTM D412-98a with a stretching rate at 100 mm/min. The tensile tests were conducted for five times on an average.

### 2.4. Biocompatibility Test

Biocompatibility tests were performed by using the ISO 10993-5 regulation through Alamar Blue assay and lactate dehydrogenase (LDH) assay [34]. Alamar Blue assay was carried out according to the following steps: first, 3T3 cells were cultured under 10% fetal bovine serum (FBS) Dulbecco’s modified eagle’s medium (DMEM) at 37 °C in the presence of 5% CO_2_. All Cells were subcultured twice before the following experiment. In the next step, the specimens were polymerized under photo-polymerization machine on top of a round Teflon mold, which is 8 mm in diameter and 2 mm in height, in a distance of 0.5 cm for 40 s. After the polymerization, the UA composites were then removed from the mold, sterilized by UV-light for 24 h, and soaked in fresh medium at 37 °C for 24 h. In the final step, the medium containing 3T3 cells were added to 96-well plates with a cell number of 104/well. After the cells fully adhered on the plate, the original cell culture medium was replaced by the extract material. The cell incubation for Alamar blue assay cell activity analysis was conducted at 37 °C for 1, 3, and 7 days in the presence of 5% CO_2_. Moreover, the evaluation of LDH assay for cytotoxicity were also conducted for 1, 3, and 7 days by using 3T3 cells under similar culture condition in 10% FBS DMEM at 37 °C in the presence of 5% CO_2_.

### 2.5. Antibacterial Test

**Figure 4:**
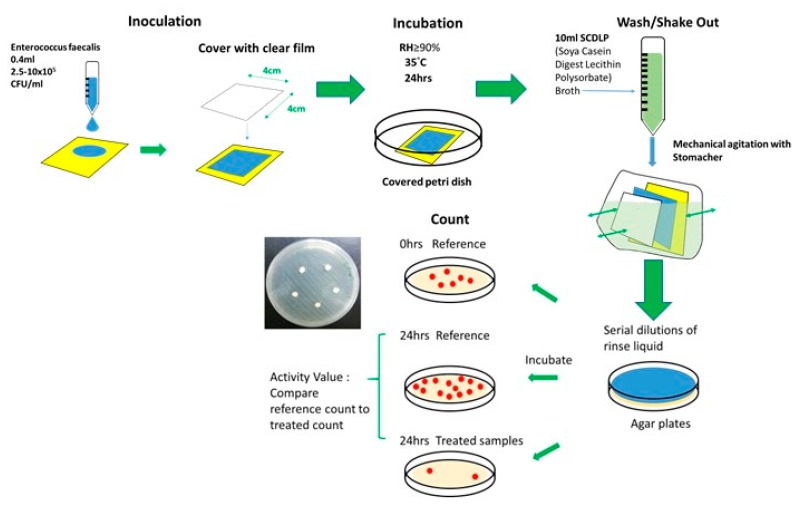
The process of antibacterial evaluation.

The antibacterial activity of UAT65/TPGDA or UAC50/TPGDA with various Ag@NSP, ZnO@NSP, or Ag/ZnO@NSP contents was performed according to the Japanese Industrial Standard as shown in Figure 4 (JIS Z 2801-2000) [32]. Gram-positive *E. faecalis* (ATCC 29212, Super Laboratory Co., Taiwan) was the bacterial strain cultivated in brain heart infusion (BHI) broth. The number of bacterial suspensions was adjusted to 104 colony-forming units per milliliter (CFU/mL). The antibacterial test was carried out by the following steps. First, colonies were added into 5 mL HBI broth, cultivated at 37 °C for 16–18 h. The medium of bacteria fluid of lysogeny broth (LB) was replaced by phosphate-buffered saline (PBS) by using the centrifugation for three times (8,000 xg, 3 min), and diluted to the concentration of 10^−5^–10^−7^ (CFU/mL). Subsequently, the as-prepared suspension was spread onto the BHI agar plates (10 μL) and incubated cells at 37 °C for 16–18 h. The 0.5 McFarland (10^8^ CFU/mL) bacterial fluid was produced by using *E. faecalis* (ATCC 29212). Specimens were shaped to square (5 cm × 5 cm), wiped with alcohol, and sterilized by the exposure of UV-light for 24 h. Then, 400 μL bacterial fluid (10^4^ CFU/mL) was added to the specimens, covered with a sterile square PE film (4 cm × 4 cm) and incubated at 37 °C under relative humidity 90% for 24 h. After bacterial adhesion or proliferation, the specimens were rinsed for three times by using PBS and then transformed to a new 24-well plate. Each specimen was soaked in 2.5% glutaraldehyde and reacted at 4 °C for 1 h, followed by the removal of glutaraldehyde by rinsing with PBS for three times. Before the investigation of morphology for the evaluation of antibacterial activity, the samples were prepared by using ethanol-wet bonding technique [35]. The freeze-drying processes were conducted for 24 h by the increased concentration of (*w/w*) ethanol from 30%, 50%, 70%, 90%, 95%, 99% to 100%. In addition, the statistical analysis was performed by Statistical Analysis Software (SAS). One-way analysis of variance (ANOVA) was used to analyze the difference between bacterial groups, and Duncan’s multiple tests were used to distinguish various bacterial groups. The *p*-value <0.05 is regarded as statistically significant.

## 3. Results and Discussion

### 3.1. Synthesis of Urethan-acrylate (UA)

**Figure 5:**
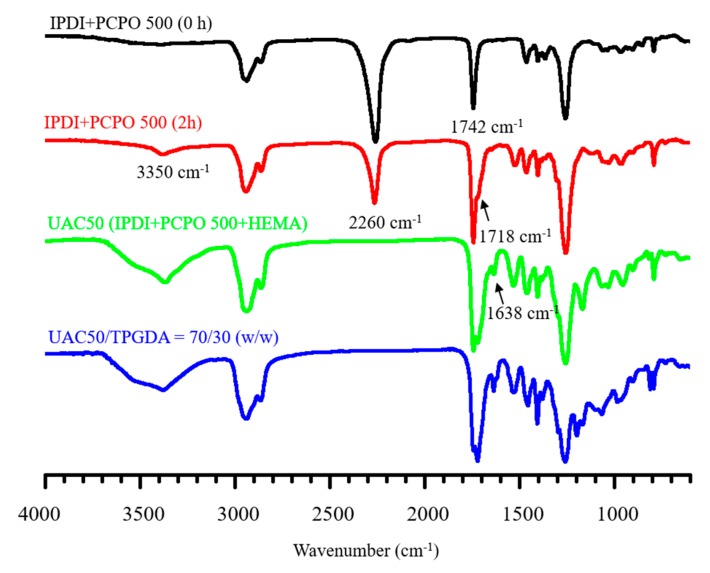
FTIR spectra of UAC50 and UAC50/TPGDA.

The preparation of urethane acrylate was monitored by using the IR spectra as shown in Figure 5. For the spectrum of the initial mixture of IPDI and PCPO 500, two distinct peaks at 2260 and 1742 cm^−1^ were present for isocyanate, and carbonyl group of carbonate ester, respectively. After 2 h into the reaction, a newly emerged shoulder at 1718 cm^−1^ was observed, indicating the formation of urethane carbonyl group [36,37]. Moreover, the urethane prepolymer end-capped with isocyanates was further reacted with HEMA to provide the product of UAC50 as evident by the near disappearance of isocyanate group and the formation of a new adsorption peak at 1638 cm^−1^ of vinyl group. The additional TPGDA in the mixture of UAC50/TPGDA = 70/30 (*w/w*) resulted in a stronger absorption intensity of vinyl group. The weight average molecular weights were measured by using GPC, leading to the results of 3320 g/mol for UAC50 and 3620 g/mol for UAT65.

### 3.2. Preparation and Thermal Properties of UA Composites

**Figure 6:**
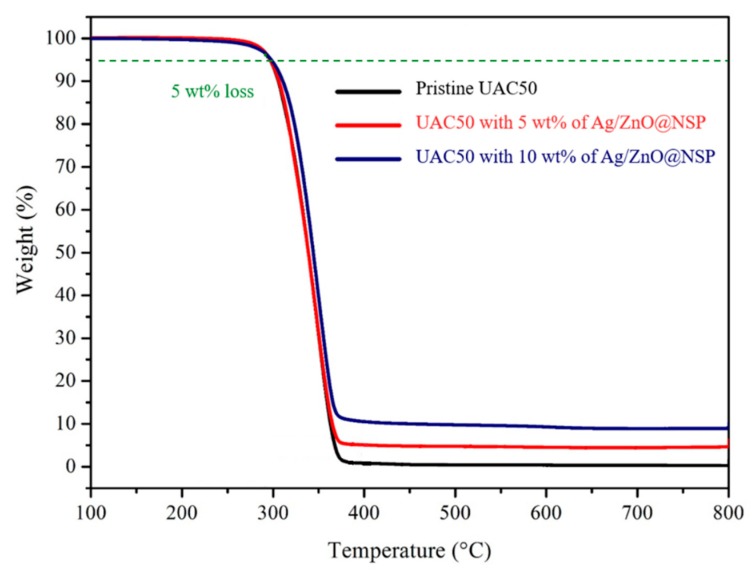
Thermogravimetric analysis (TGA) thermograms of UAC50- and UAC50-based composites.

The determination of the processing effectiveness and the quantitative fillers within the matrix are usually investigated by using thermogravimetric analysis (TGA) for the polymer composites [38]. The UA composites were prepared by mixing 5 wt % or 10 wt % in total composites by using the nanometer-thin silicate platelets immobilized with nanoparticles such as AgNPs (Ag@NSP), ZnONPs (ZnO@NSP), or AgNPs together with ZnONPs (Ag/ZnO@NSP) [20,22,25,39,40]. In TGA thermograms (Figure 6), the UA composites exhibited 5% weight loss at about 300 °C, indicating that good thermal stability was available for the following tests. Moreover, the addition of nanoparticles immobilized on the nanometer-thin silicate platelets provided higher char yield (%) dependent on the addition of inorganic contents.

### 3.3. Curing Conditions and Depths for UA Composites

**Figure 7:**
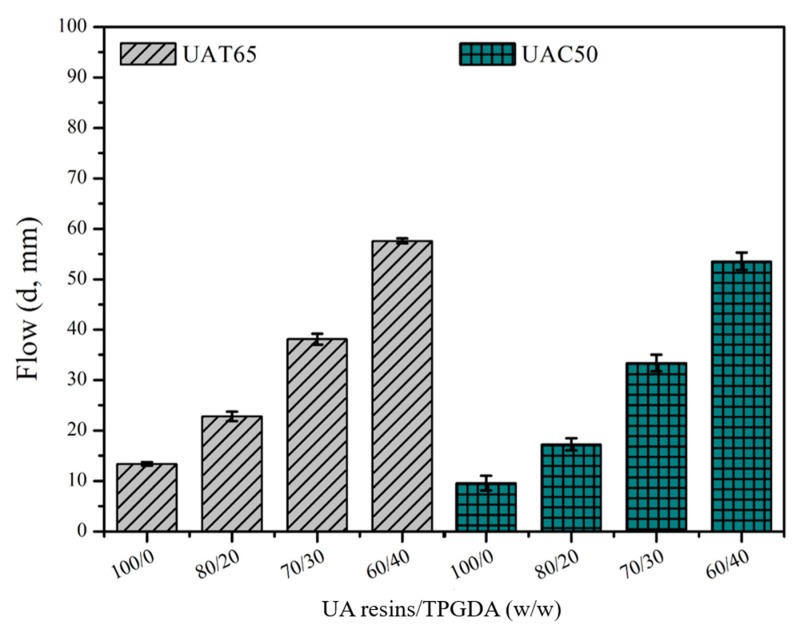
Flow analysis of UA resins with various weight ratios of UAT65/tripropylene glycol diacrylate (TPGDA) or UAC50/TPGDA according to ISO 4049:2009.

The endodontic sealers are required to exhibit several properties to meet the desired performance. The flow behavior is one of the most important factors for sealers to penetrate into small irregularities of the root canal system and dentinal tubules as shown in Figure 7. According to the regulation of ISO 4049:2009, the flow diameter should be over 20 mm after compression under a weight disc for 10 min. The tests were carried out by the introduction of difunctional TPGDA in order to increase flow diameter and crosslinking density. As a result, the samples with higher ratio of TPGDA exhibited larger flow diameter, such as UAT65/TPGDA = 80/20, 70/30, or 60/40; or UAC50/TPGDA = 70/30 or 60/40, since the difunctional TPGDA monomer is a diluent with a lower molecular weight when compared with the UA resins.

**Figure 8:**
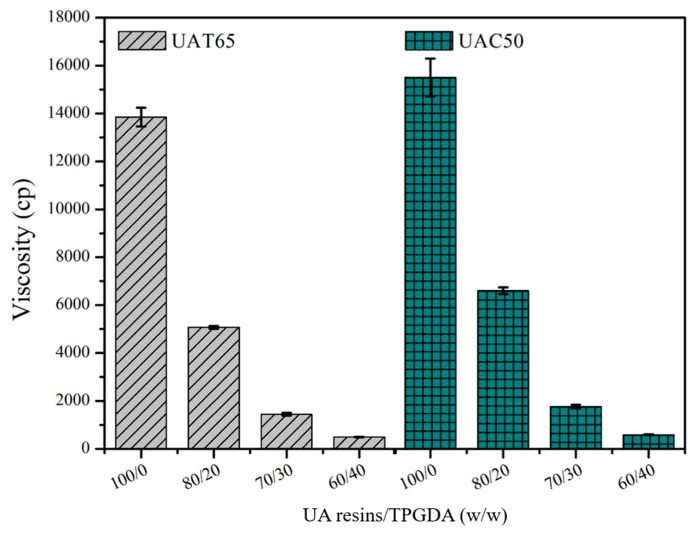
Viscosity analysis of UA resins with various weight ratios of UAT65/TPGDA or UAC50/TPGDA according to ISO 4049:2009.

Moreover, viscosity is another important factor for the root canal sealer to establish connection between the root canal, periodontal ligament, and the apical foramen. According to ISO 4049:2009, the viscosity should range from 1000 to 2000 cp to meet the desired operation [32]. In Figure 8, UAT65/TPGDA (70/30) and UAC50/TPGDA (70/30) were the samples of choice for further investigations since the addition of the optimized TPGDA content would achieve the viscosity range mentioned above. It is important to note that flow and viscosity properties depends on not only the ratios between UA resins and TPGDA but the use of polyols such as PTMEG or PCPO. The PCPO-based UA resins exhibited somewhat lower flow diameter and higher viscosity under the same ratio of UA/TPGDA composition. This is because the incorporation of the PCPO-based polyol with carbonate groups brought about more hydrogen bonding interactions with the urethane linkages. This would restrain the molecular mobility of the UA segments [41,42].

The curing conditions of UAT65/TPGDA (70/30) or UAC50/TPGDA (70/30) were carried out by using ternary initiation processes for photo crosslinking. The degree of acrylate conversion (DC%) would be based on the peak area at 1638 cm^−1^ of aliphatic C=C double bond of acrylate content as a function of various initiator concentrations (1, 3, 6, and 9 phr) before and after curing (Table 1). Optimum mechanical properties with a tensile strength of 56.15 ± 3.26 MPa and a Young’s modulus of 361.88 ± 32.38 MPa were achieved for the sample UAT65/TPGDA (70/30) cured with 3 phr initiator concentration. It is important to note that the DC% values were close to 70% for the above-mentioned samples. This is because the low conversion degree of acrylate functional groups with a low concentration initiator resulted in insufficient crosslinking density for obtaining good mechanical performance, whereas the excessive photoinitiators would provide the localized absorption and crosslinking density over the percolation threshold, leading to the adverse effect on the mechanical properties [43].

**Figure 9:**
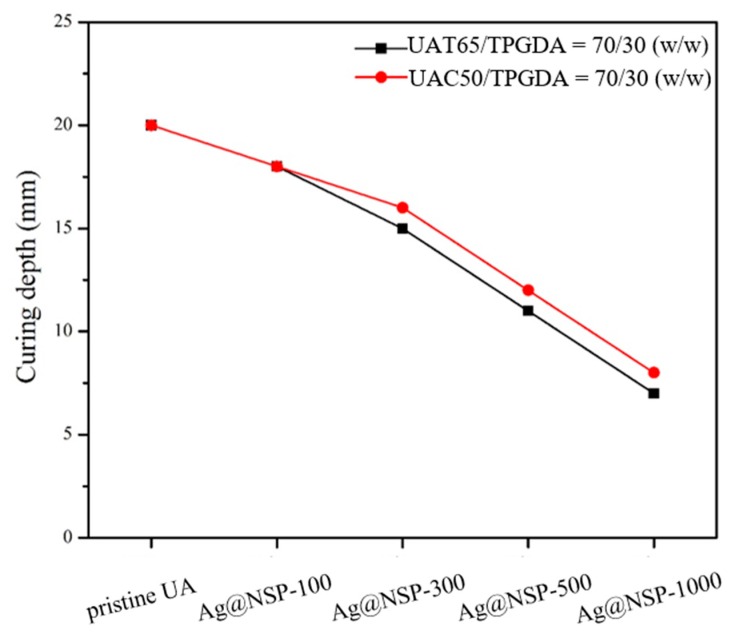
Curing depths of UAT65/TPGDA resins (*w/w* = 70/30) and UAC50/TPGDA resins (*w/w* = 70/30) with various Ag@NSP contents (ppm).

**Figure 10:**
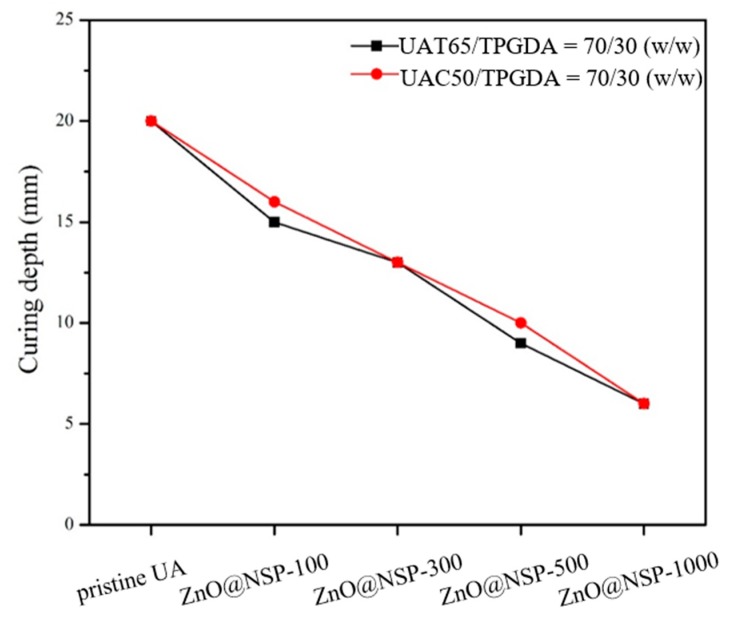
Curing depths of UAT65/TPGDA resins (*w/w* = 70/30) and UAC50/TPGDA resins (*w/w* = 70/30) with various ZnO@NSP contents (ppm).

**Figure 11:**
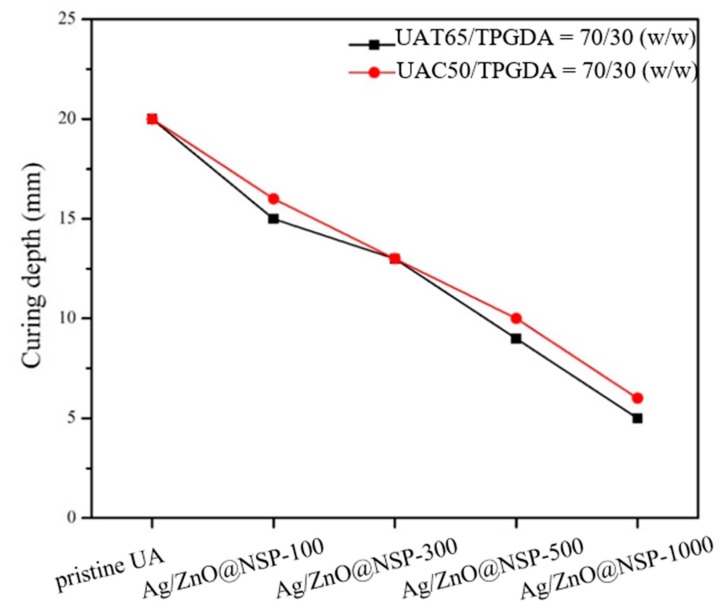
Curing depths of UAT65/TPGDA resins (*w/w* = 70/30) and UAC50/TPGDA resins (*w/w* = 70/30) with various Ag/ZnO@NSP contents (ppm).

Since the acrylate-based photopolymerization depends deeply on the sealer transparency, the curing depths of UAT65/TPGDA resin (*w/w* = 70/30) and UAC50/TPGDA resin (*w/w* = 70/30) were assessed by using various contents of antibacterial NSPs immobilized with nanoparticles including Ag@NSP, ZnO@NSP, and Ag/ZnO@NSP (Figure 9, Figure 10 and Figure 11). It is reported that the photo crosslinking activity relies on the use of different types of metal nanoparticles and the rate of photo crosslinking, and the DC% also varies with the additives under similar processing condition [44]. In this study, the curing depths decreased with increasing content of antibacterial NSP agents.

These UA composite composed of various antibacterial nanomaterials are denoted as Ag@NSP-n, ZnO@NSP-n, or Ag/ZnO@NSP-n, where the “n” is denoted as the parts per million (ppm) to the total weight of UAT65/TPGDA or UAC50/TPGDA. According to the regulation of ISO 4049:2009, the curing depth should be larger than 10 mm. As a result, the limitation for the addition of a maximum amount of Ag@NSP is 500 ppm (Ag@NSP-500) as shown in Figure 9. Similar results were also obtained for the composites incorporated with ZnO@NSP or Ag/ZnO@NSP (Figure 10 and Figure 11). This indicates that the presence of different nanomaterials such as Ag@NSP, ZnO@NSP, or Ag/ZnO@NSP in the UA composites did not influence curing depths much.

### 3.4. Biocompatibility Analysis

**Figure 12:**
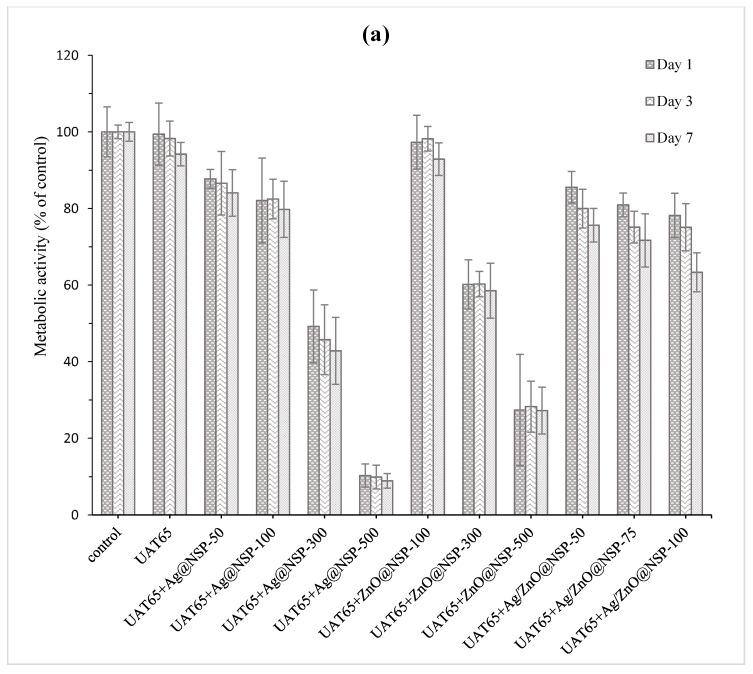

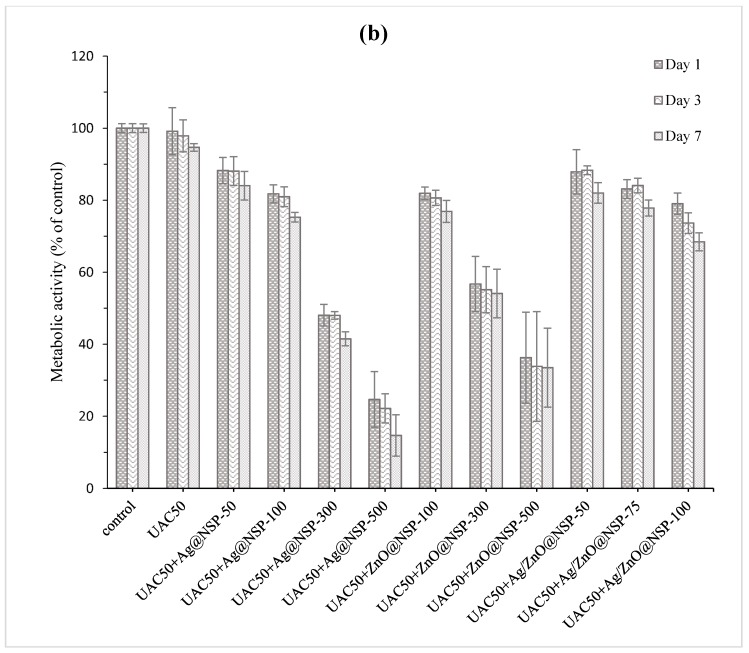
Alamar blue assay of (**a**) UAT65/TPGDA (*w/w* = 70/30) and (**b**) UAC50/TPGDA (*w/w* = 70/30) resins with various concentrations of Ag@NSP, ZnO@NSP, or Ag/ZnO@NSP (*p* < 0.05).

**Figure 13:**
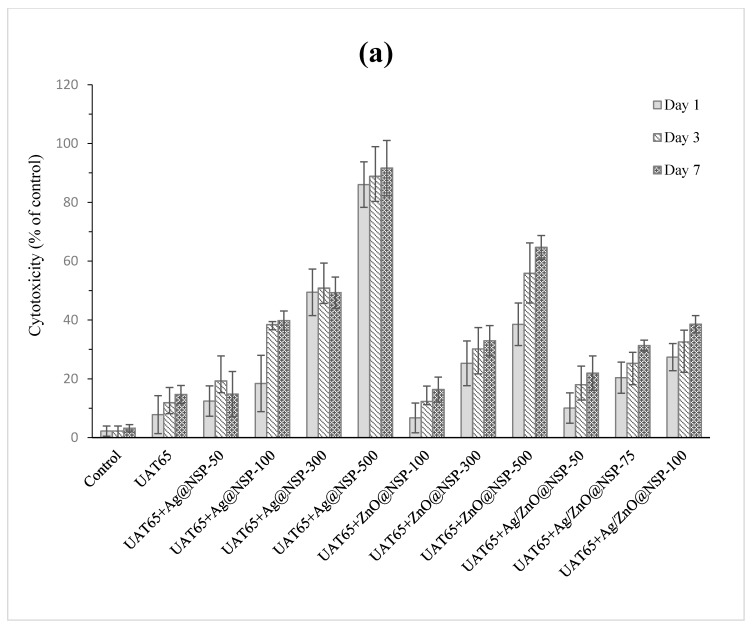

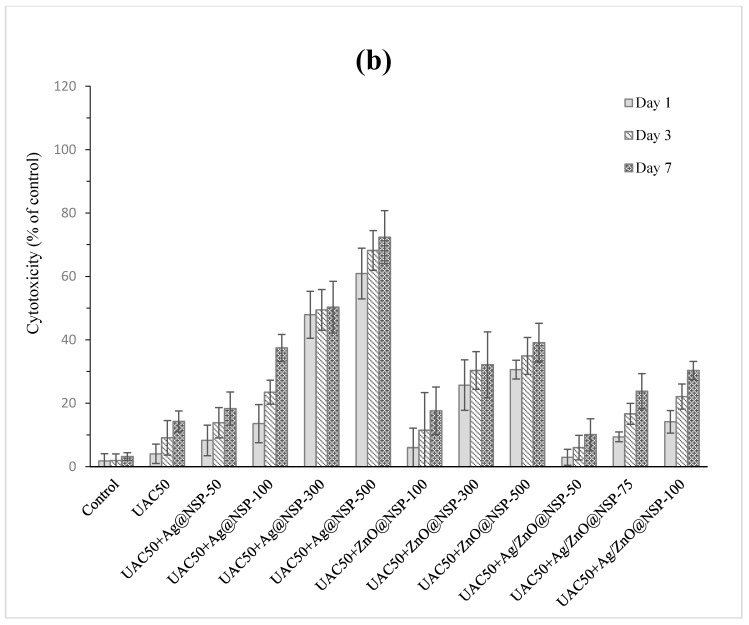
Lactate dehydrogenase (LDH) assay of (**a**) UAT65/TPGDA (*w/w* = 70/30) and (**b**) UAC50/TPGDA (*w/w* = 70/30) with various concentrations of Ag@NSP, ZnO@NSP, or Ag/ZnO@NSP (*p* < 0.05).

Grossman [45] advocated that an ideal root canal filling material should not irritate periradicular tissues. In addition, Faccioni et al. found that root canal materials with metal ions might influence cell metabolism and differentiation [46]. Other studies found that the incomplete photopolymerization reaction resulted in the release of uncured monomers and initiators, which would affect the mitochondrial enzyme activity [47,48]. Therefore, the concentration of antibacterial agent in sealers depends deeply on the biocompatibility of composites. In this study, the tests of Alamar Blue assay and LDH assay were conducted for the biocompatibility tests for UAT65/TPGDA (*w/w* = 70/30) and UAC50/TPGDA resins (*w/w* = 70/30) with various concentrations of antimicrobial agents such as Ag@NSP, ZnO@NSP, and Ag/ZnO@NSP as shown in Figure 12 and Figure 13, respectively.

In the Alamar blue assay test, both pristine resins of UAT65/TPGDA (*w/w* = 70/30) (Figure 12a) and UAC50/TPGDA (*w/w* = 70/30) (Figure 12b) were substantially free of cytotoxicity. As the antimicrobial agents were incorporated into the resins to form UA composites, reduced metabolic activities were observed, as shown in Figure 12. Furthermore, the composite with Ag@NSP exhibited the poorest biocompatibility when compared to other samples, especially in the example for both UAT65/TPGDA and UAC50/TPGDA incorporated with 500 ppm additives. The metabolic activities were higher than 70% for all the UA composites with 100 ppm or less than 100 ppm antibacterial NSP agents. This indicates that good biocompatibility could be achieved with the addition of a certain content of the antimicrobial agents such as Ag@NSP, ZnO@NSP, or Ag/ZnO@NSP to the composites.

In the LDH assay test, the cytotoxicity is also dependent on the addition of various concentrations of Ag@NSP, ZnO@NSP, and Ag/ZnO@NSP to the composites (Figure 13). Given the fact that UA composites with 100 ppm would exhibit good biocompatibility, the cytotoxicity of the UA composites with 100 ppm antimicrobial agents on 3T3 cells was investigated and observed in the following order: Ag@NSP-100 > Ag/ZnO@NSP-100 > ZnO@NSP-100. This implies that the composites comprising AgNPs would exhibit poor cytotoxicity performance. In addition, the cytotoxicity of the composites based on the UAC50/TPGDA (*w/w* = 70/30) are lower than that of the composites based on UAT65/TPGDA (*w/w* = 70/30), especially in the example for both UAT65/TPGDA (~90% of control) and UAC50/TPGDA (~80% of control) incorporated with 500 ppm Ag@NSP. As a matter of fact, polycarbonate-based polyurethanes (PUs) with better biocompatibility correspond to the weaker immune response when compared with polyether-based PUs according to the literature [49]. This is because the α-carbon atoms of the polyether-based PUs (such as UAT65) are highly susceptible to oxidation by oxygen radicals to form esters, which result in unstable chemical structures for polymers [50]. As a result, the carbonate containing UAC50/TPGDA system would be the material of choice for dental root canal sealers instead of the ether-containing UAT65/TPGDA resins.

### 3.5. Antibacterial Analysis

The antibacterial activities of the composites based on UAC50/TPGDA (*w/w* = 70/30) with nanoparticles-on-platelet nanohybrids (Ag@NSP, ZnO@NSP, Ag/ZnO@NSP) were analyzed by treating the composites with Gram-positive *E. faecalis* (ATCC 29212) according to Japanese Industrial Standard (JIS Z 2801-2000) as shown in Table 2. According to the biocompatibility tests in previous section, the use of antibacterial NSP agents should not be higher than 100 ppm for UAC50/TPGDA (*w/w* = 70/30) resins. Indeed, the AgNPs-based composites with 75 ppm (Ag@NSP-75) or 100 ppm (Ag@NSP-100) inorganic additives exhibited antibacterial activity. However, ZnONPs-based composites even with 1000 ppm (ZnO@NSP-1000) were not able to exhibit antibacterial activity. In fact, the antibacterial activity was feasible only when the use of high concentration of ZnONPs over 2000 ppm (ZnO@NSP-2000 and ZnO@NSP-3000), indicating that the Ag@NSP-based composites would be better candidates for the root canal sealer applications. In addition, the simultaneous immobilization of AgNPs and ZnONPs on silicate platelets, i.e., Ag/ZnO@NSP, led to much better antimicrobial results even for the composite with an antibacterial agent concentration as low as 50 ppm (Ag/ZnO@NSP-50).

**Figure 14:**
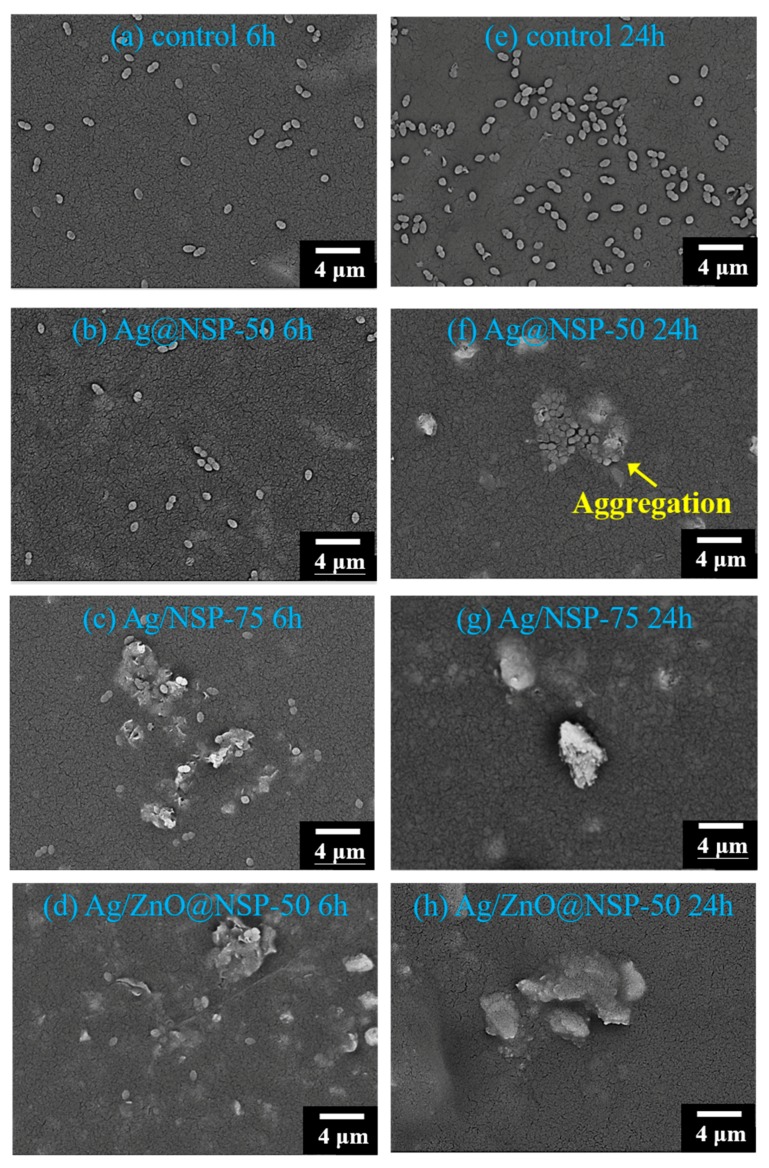
SEM images (2500x) of *E. faecalis* on the surfaces of UAC50/TPGDA = (*w/w* = 70/30) with various antibacterial agents: control (**a**,**e**); 50 ppm Ag@NSP (**b**,**f**); 75 ppm Ag@NSP (**c**,**g**); 50 ppm Ag/ZnO@NSP (**d**,**h**) for 6 h and 24 h, respectively.

The direct contact of UAC50/TPGDA composites with the bacterial population could be visualized by using scanning electron microscopy (SEM) as shown in Figure 14. According to the investigation above, this study was conducted by using *E. faecalis* on the surfaces of UA/TPGDA composites with 50 ppm Ag@NSP, 75 ppm Ag@NSP, and 50 ppm Ag/ZnO@NSP after 6h and 24h. For the UAC50/TPGDA resin without the use of antibacterial agents, a rapid growth of bacterial in number was observed as shown in Figure 14a,e. For the composite (Ag@NSP-50) with 50 ppm of Ag@NSP, a certain amount of bacteria appeared first after 6h (Figure 14b). Subsequently, these bacteria increased in number and aggregated after 24 h (Figure 14f). For the composite (Ag@NSP-75) with 75 ppm of Ag@NSP, a certain amount of bacteria appeared to be aggregated and deformed after 6h, and subsequently these bacteria remained aggregated without the sign of number increase as shown in Figure 14c,g, respectively. It is likely that the nanoparticles can re-charge from NSPs to inactivate and rupture bacterial aggregates [51].

It was reported that the combined use of different types of nanoparticles could adsorb onto the cytoderm of the bacteria and even penetrate the cytomembrane to disturb the normal function of cells, leading to cell apoptosis [50]. This is evidenced by the presence of only 50 ppm Ag/ZnO@NSP in the UAC50/TPGDA composite capable of exhibiting a satisfactory antibacterial effect against *E. faecalis* (Table 2). For the composites (Ag/ZnO@NSP-50) with 50 ppm of Ag/ZnO@NSP, once again a certain amount of bacteria appeared to be aggregated and deformed after 6h, and subsequently these bacteria remained aggregated without the sign of number increase as shown in Figure 14d,f. The simultaneous immobilization of AgNPs and ZnONPs on silicate platelets could not only enhance the antibacterial activities and reduce the dose of AgNPs, but act as a promoter in the antibacterial effect for the Ag/ZnO@NSP-based composites as well.

## 4. Conclusions

The purpose of this study is to develop a new root canal sealer with a biocompatible resin based on a macrodiol of polycarbonate (PCPO) that could be prepared through the utilization of carbon dioxide as feedstock. With good biocompatibility and biostability, these carbonate-containing urethane acrylate resins would be able to exhibit superior performance to the reference, polyether (PTMEG)-based resins in the preparation of root canal obturation sealers. The successful incorporation of nanoparticles immobilized on nanoscale platelets in the resin matrix resulted in the root canal obturation sealers with satisfactory biocompatibility and antibacterial effect. As a result, the PCPO-based urethane acrylate was selected to be the resin sealer matrix. Moreover, the incorporation of ZnONPs and AgNPs simultaneously immobilized on silicate platelets into the PCPO-based urethane acrylates would not only enhance the antibacterial activities, but also serve as a promoter in the antibacterial effect. Based on the above, the UAC50/TPGDA (70/30 = *w/w*) resin with 50 ppm Ag/ZnO@NSP has a great potential as an antibacterial root canal sealer.

## Figures and Tables

**Table 1:** Mechanical properties and DC% for the UAT65/TPGDA (*w/w* = 70/30) and TPGDAC50/TPGDA (*w/w* = 70/30) samples after curing with different dosages of photoinitiator.

Entry	UA Formulation	Photoinitiator (phr ^1^)	Tensile Strength (MPa)	Young’s Modulus (MPa)	DC ^2^ (%)
1	UAT65/TPGDA (*w/w* = 70/30)	1	51.71 ± 3.03	362.81 ± 37.10	46.27 ± 3.07
2	UAT65/TPGDA (*w/w* = 70/30)	3	56.15 ± 3.26	361.88 ± 32.38	64.91 ± 1.06
3	UAT65/TPGDA (*w/w* = 70/30)	6	45.96 ± 2.22	33.49 ± 1.65	67.58 ± 2.27
4	UAT65/TPGDA (*w/w* = 70/30)	9	36.78 ± 1.65	1.92 ± 0.42	70.35 ± 1.76
5	UAC50/TPGDA (*w/w* = 70/30)	1	46.30 ± 3.18	1072.25 ± 46.54	57.89 ± 0.41
6	UAC50/TPGDA (*w/w* = 70/30)	3	60.54 ± 4.72	1042.02 ± 39.62	72.44 ± 1.57
7	UAC50/TPGDA (*w/w* = 70/30)	6	61.09 ± 1.15	14.01 ± 2.65	79.03 ± 2.57
8	UAC50/TPGDA (*w/w* = 70/30)	9	59.04 ± 2.75	8.61 ± 0.41	80.08 ± 2.79

^1^: parts per hundred; ^2^: after the exposure under UV light for 40 s.

**Table 2:** Antibacterial activity of the composites based on UAC50/TPGDA (*w/w* = 70/30) resins with various concentrations of Ag@NSP, ZnO@NSP, or Ag/ZnO@NSP.

Entry	Type of Composites ^(1)^	Antibacterial Activity ^(2)^
1	Ag@NSP-50	╳
2	Ag@NSP-75	○
3	Ag@NSP-100	○
4	ZnO@NSP-100	╳
5	ZnO@NSP-200	╳
6	ZnO@NSP-300	╳
7	ZnO@NSP-500	╳
8	ZnO@NSP-1000	╳
9	ZnO@NSP-2000	○
10	ZnO@NSP-3000	○
11	Ag/ZnO@NSP-10	╳
12	Ag/ZnO@NSP-25	╳
13	Ag/ZnO@NSP-50	○
14	Ag/ZnO@NSP-75	○
15	Ag/ZnO@NSP-100	○

(1): composites using UAC50 as polymer matrix; (2): “○“ indicating the inhibition of bacteria; “╳“ indicating the growth of bacteria.
